# Supplementary material for: Modified relaxation dynamics and coherent energy exchange in coupled vibration-cavity polaritons
Source: Nat Commun. 2016 Nov 22;7:13504. doi: 10.1038/ncomms13504 (PMC5121416; doi:10.1038/ncomms13504)
Supplement: Supplementary Information — Supplementary Figures 1-5, Supplementary Tables 1-3, Supplementary Notes 1-6 and Supplementary References [file ncomms13504-s1.pdf]

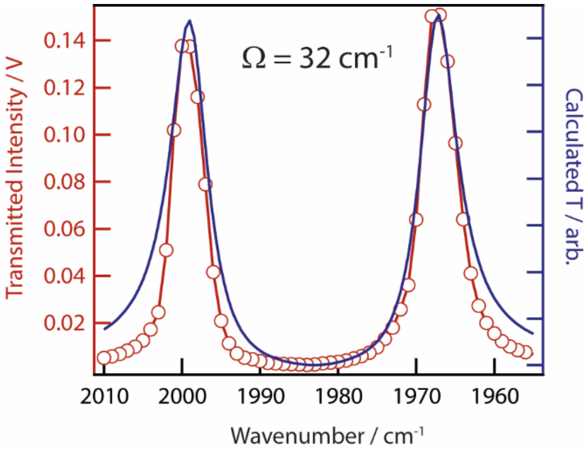

Supplementary Figure 1 | Experimental (red points) and calculated (blue trace) transmission spectra of 20 mM $W(CO)_6$ in hexane in a 25 μm cavity angle tuned for the strongest coupling, *i.e.*, smallest observed splitting, between the C-O asymmetric stretch at 1983 cm^{-1} and a cavity mode.

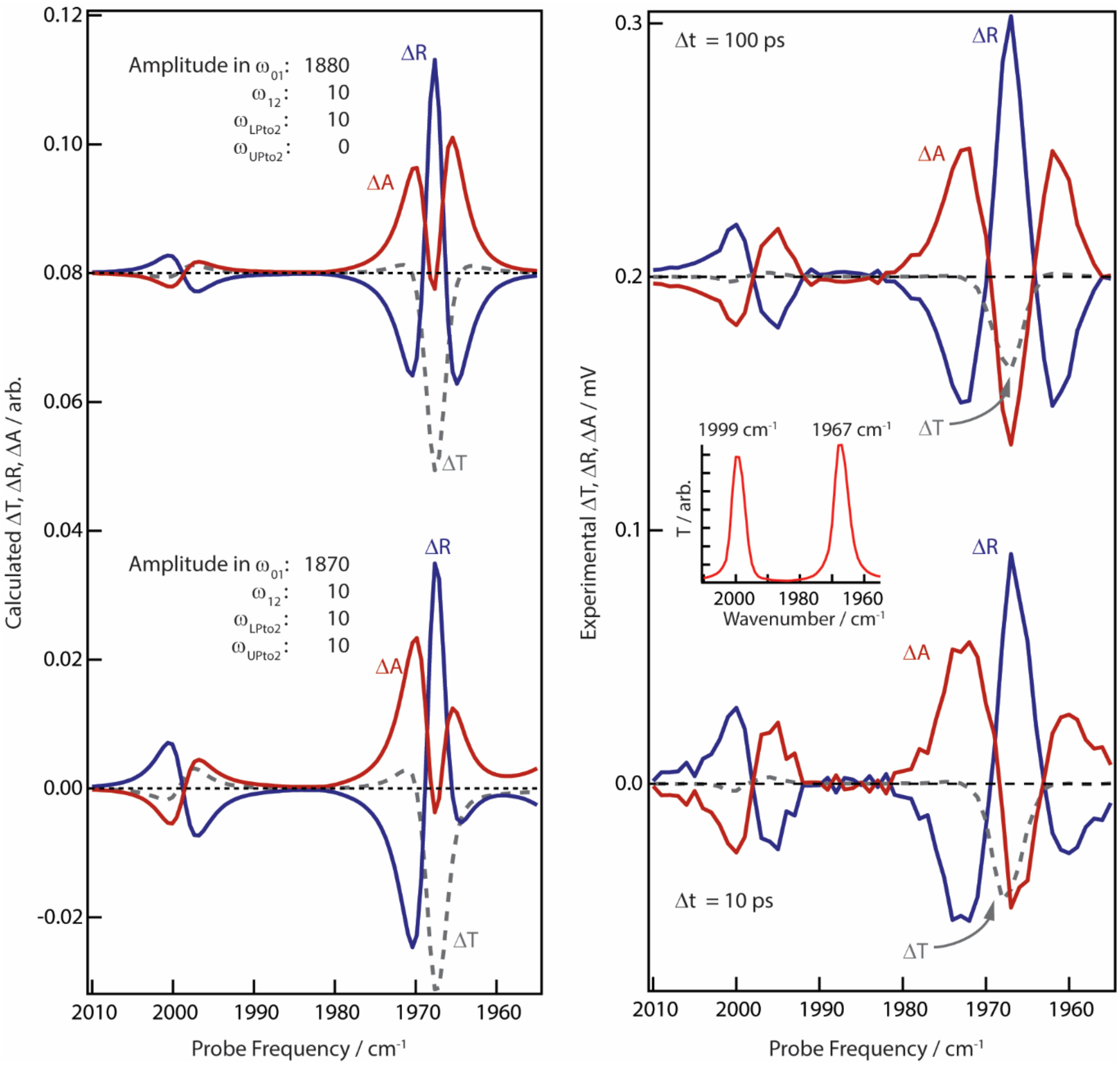

Supplementary Figure 2 | Calculated (left) and experimental (right) differential reflection (solid blue), transmission (dashed grey), and absorption (solid red) spectra of 20 mM $W(CO)_6$ in hexane coupled to a cavity. The experimental curves were measured 10 ps (bottom right) and 100 ps (top right) after excitation. Lorentzian amplitudes are in units of cm^{-2}. To calculate the spectra, we computed excited-state spectra with the population distributions described in the text and subtracted the ground-state spectrum from excited-state spectra shown in Supp. Fig. 2.

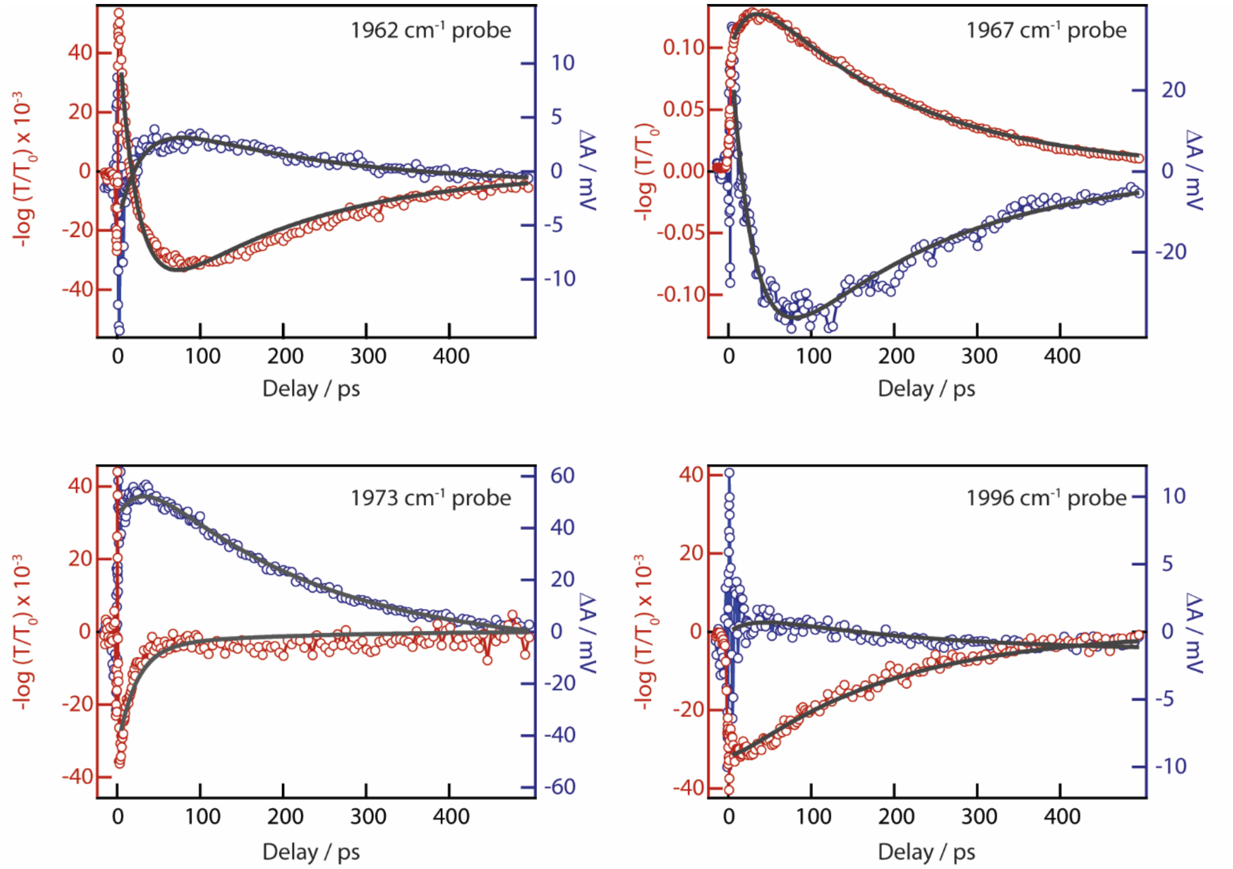

Supplementary Figure 3 | Kinetic traces plotted in units of ΔA (blue) and $-\log_{10}(T/T_0)$ (red) at probe frequencies where ΔA is large. Each set of four traces was globally fit to a biexponential decay (fit results are solid lines), *i.e.*, one global fit for ΔA, one global fit for $-\log_{10}(T/T_0)$.

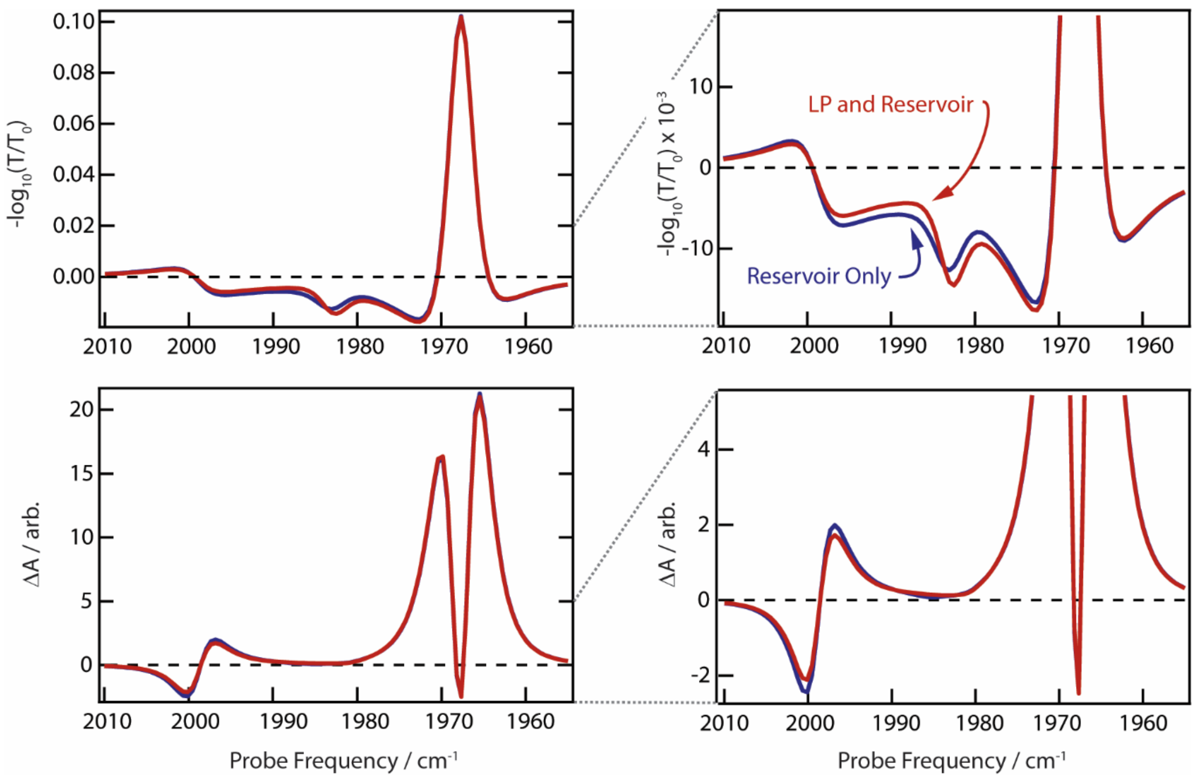

Supplementary Figure 4 | Calculated $-\log_{10}(T/T_0)$ (top) and ΔA (bottom) spectra for excited-state population distributions including LP and reservoir v = 1 population (red) and only reservoir v = 1 population (blue). The Lorentzian amplitudes, in units of cm^{-2}, used are A_{01} = 1880, A_{12} = 10, and A_{LP} to 2 = 10 for the red traces and A_{01} = 1890 and A_{12} = 10 for the blue traces. The right panels are magnified views of the left panels.

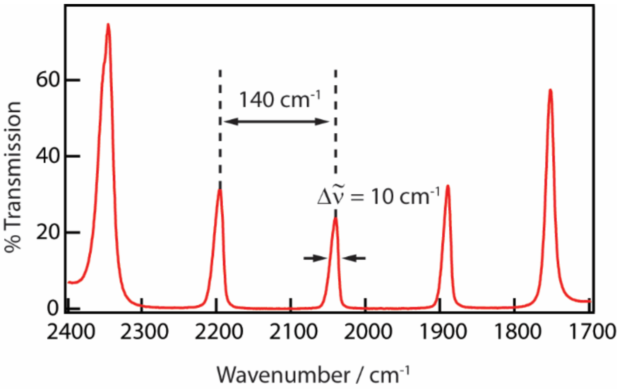

Supplementary Figure 5 | FTIR spectrum of empty cavity comprising dielectric mirrors separated by nominally 25 μm PTFE spacer.

Supplementary Table 1 | Global Fit Parameters for Supp. Fig. 3

Global fit parameters for the fits shown in Fig. S4. Time constants are constrained to be the same for each group of measurements (*i.e.*, ΔA and $-\log_{10}(T/T_0)$) and obtained from biexponential decays. Fitting ΔA requires a small constant offset, designated ΔA_0.

Probe Frequency / cm^{-1}		τ_{UP} / ps	τ_{slow} / ps	A_{UP}	A_{slow}	ΔA_0
$-\log_{10}(T/T_0)$	1962	23 ± 3	190 ± 25	-0.001	-0.011	
	1967			0.096	-0.062	
	1973			-0.030	0.079	
	1996			-0.002	0.003	
ΔA	1962	28 ± 4	200 ± 30	0.110	-0.055	-0.001
	1967			-0.078	0.173	0
	1973			-0.029	-0.004	-0.007
	1996			0.003	-0.035	-0.001

Supplementary Table 2 | Global Fit Parameters for Fig. 4

Global fit parameters for the fits shown in main text Fig. 4. Time constants are constrained to be the same for each probe frequency.

Probe Frequency / cm^{-1}	τ_{UP} / ps	τ_{slow} / ps	A_{UP}	A_{slow}
1961	22 ± 3	170 ± 25	0.040	-0.012
1970			-0.089	0.19
1976			0	-0.062
1994			0.004	-0.017
2004			-0.002	0.010

Supplementary Table 3 | Global Fit Parameters for Angle-Tuned Kinetics

Global fit parameters for the fits shown in main text Fig. 6c. To obtain time constants, we measured the decay dynamics for each incidence angle at three different probe frequencies. For each incidence angle, time constants are constrained to be same for each probe frequency.

Probe Frequency / cm^{-1}	τ_{UP} / ps	τ_{slow} / ps	A_{UP}	A_{slow}
1966, 29°	21 ± 3	200 ± 30	-0.10	0.15
1968, 29°			-0.14	0.18
1970, 29°			-0.15	0.18
1963, 27°	50 ± 7	160 ± 20	-0.07	-0.12
1966, 27°			-0.14	0.21
1968, 27°			-0.13	0.17

Supplementary Note 1: Modeling of Lorentzian Oscillators

Equation S1, below, is the classical equation for the transmission of a Fabry-Pérot cavity. This expression provides the basis for relating transient spectra to excited and ground state populations.

$$T_{cav}(\bar{\nu}) = \frac{T^2 e^{-\alpha L}}{1+R^2 e^{-2\alpha L}-2Re^{-\alpha L}\cos(4\pi n L\bar{\nu}+2\varphi)} \tag{S1}$$

This relationship is based on the frequency dependent absorption coefficient (α) and refractive index (n) of the material within the cavity. We obtain α and n by modeling the dielectric function of the cavity load as a sum of Lorentzian oscillators. The real and imaginary components of the dielectric function, ε_1 and ε_2, are defined as a sum of i Lorentzian oscillators according to

$$\varepsilon_1 = {n_{bg}}^2 + \sum_i \frac{A_i({\nu_{0i}}^2-\nu^2)}{({\nu_{0i}}^2-\nu^2)^2+(\Gamma_i\nu)^2} \tag{S2}$$

$$\varepsilon_2 = \sum_i \frac{A_i\Gamma_i\nu}{({\nu_{0i}}^2-\nu^2)^2+(\Gamma_i\nu)^2} \tag{S3}$$

where n_{bg} is the background refractive index, A_i the amplitude, ν_{0i} the resonant frequency, and Γ_i the full linewidth associated with the ith oscillator. The frequency-dependent refractive index, n, and absorption coefficient, α, can be formulated as

$$n = \sqrt{\frac{\varepsilon_1+\sqrt{{\varepsilon_1}^2+{\varepsilon_2}^2}}{2}} \tag{S4}$$

$$\alpha = 4\pi\nu k = 4\pi\nu\sqrt{\frac{-\varepsilon_1+\sqrt{{\varepsilon_1}^2+{\varepsilon_2}^2}}{2}} \tag{S5}$$

Initial values of A_i, ν_{0i}, and Γ_i are chosen to be consistent with the optical response of witness samples, *i.e.*, absorbance for the concentration and pathlengths used. Expressions S2 and S3 are then substituted into S4 and S5 which are substituted into S1 giving the transmission spectrum through a Fabry-Pérot cavity with tailorable parameters describing the oscillator resonance frequencies, linewidths, and amplitudes.

We use 92% for the reflectivity of the mirrors and 8% for the transmission. To simulate 20 mM $W(CO)_6$ in hexane, we use A = 1900 cm^{-2}. This value gives uncoupled absorbance and cavity-coupled transmission spectra that match our experimental results. We modify A for each oscillator to calculate spectra for different distributions of absorber (excited and ground state) concentrations. We use Γ = 6 cm^{-1} to match the linewidth measured on the ultrafast instrument, which has approximately 3 cm^{-1} resolution. Supp. Fig. 1 shows the experimental transmission spectrum and spectrum calculated using these parameters.

When calculating transient spectra, we assume harmonic oscillator behavior in absorption intensities, *i.e.*, that the absorption intensity increases with increasing final vibrational quantum number.

Supplementary Note 2: Differential Reflection and Absorption

Schwartz, *et al.* found that measuring the differential absorption (by computing it from the measured differential transmission and reflection, $\Delta A = -(\Delta T + \Delta R)$) from a system comprising dye molecules strongly coupled to a cavity was crucial for obtaining accurate kinetic information.[1] In their case, strong signals from uncoupled ground-state transitions dominated the $-\log_{10}(T/T_0)$ response that is typically reported in the ultrafast literature. Comparing ΔA to $-\log_{10}(T/T_0)$ revealed a twofold increase in half-life in the cavity compared to outside a cavity.[1]

In light of this result, we also measured the differential reflection (ΔR) from cavity-coupled $W(CO)_6$ and computed the differential absorption, ΔA. We calculate the differential responses by including the oscillators described above in a transfer-matrix computation. Although Eq. S1 does accurately predict transmission spectra, a transfer-matrix approach, which uses a system of characteristic matrices, each describing a single material layer, to calculate the response of the entire multilayer system, is necessary to model reflectivity and, therefore, absorption.[2] Supp. Fig. 2 shows calculated (left) and experimental (right) ΔT (dashed grey), ΔR (solid blue), and ΔA (solid red) spectra for 20 mM $W(CO)_6$ in hexane coupled to a Fabry-Perot cavity. We point out that the ΔT response is inverted from the traces in the main text because the main text reports $-\log_{10}(T/T_0)$.

Just as in the main text, we include UP, reservoir, and LP excited-state population to achieve agreement with the early-time experimental spectra. For this specific sample, the concentration and angle dictate that $\omega_{LP\ to\ 2}$ is 1984 cm^{-1} and $\omega_{UP\ to\ 2}$ is 1952 cm^{-1}. The calculated spectra for only reservoir and LP excited-state population match the late-time spectra. The intensities chosen and reported in Supp. Fig. 2 represent a very small (~1% total excitation) change in absorber population. The calculated spectra agree qualitatively with the experimental spectra, but seem to underestimate the differential transmission (compare ratio between ΔR and ΔT in experimental and calculated spectra). We speculate that broadband absorptive loss in the dielectric mirror coating could account for this discrepancy.

We also evaluate the kinetic response of the differential absorption and compare it to the results obtained from fitting kinetic traces in units of $-\log_{10}(T/T_0)$. Supp. Fig. 3 shows four comparisons between ΔA and $-\log_{10}(T/T_0)$, chosen at probe frequencies where ΔA is large.

To compare the kinetics, we globally fit each set of four decays to the same biexponential decay outlined in the main text. For this particular solution and angle of incidence (approximately zero detuning, $\Omega = 32$ cm^{-1}, transmission spectrum in Supp. Fig. 1), we obtained 23±3 ps for the UP decay and 190±25 ps for the slow decay from the $-\log_{10}(T/T_0)$ kinetics (paramters in Supp.

Table 1). The global fit to the ΔA kinetics yielded values of 28±3 ps for the UP decay and 200±25 ps for the slow decay.

The results from ΔA or $-\log_{10}(T/T_0)$ agree to within the uncertainties and indicate that the UP lifetime, based on the 26.5 ps average of the $-\log_{10}(T/T_0)$ and ΔA fits, is much shorter than the uncoupled v = 1 lifetime of 140 ps. The transient spectra for $W(CO)_6$ show no evidence for anomalous signals at certain probe frequencies that might distort the kinetic analysis, as observed by Schwartz, *et al*.[1] These results point to the importance of consistently using the same measurement, *i.e.*, ΔA or $-\log_{10}(T/T_0)$, when comparing dynamics at different concentration or angles of incidence.

Supplementary Note 3: Choice of Oscillators and Implications for Model

Calculating the ground-state spectrum is relatively straightforward. Oscillators at ω_{01} couple to the cavity field, giving rise to two transmissive features. After population evolves in the excited states, we then calculate a snapshot of the transmission of the non-equilibrium population in the cavity. We reduce the Lorentzian amplitude at ω_{01} to account for the reduced population in v = 0 and include oscillators at ω_{12} = 1968 cm^{-1}, $\omega_{\mathrm{LP\ to\ 2}}$ = 1984 cm^{-1}, and $\omega_{\mathrm{UP\ to\ 2}}$ = 1951 cm^{-1} to account for increased population in v = 1, LP, and UP. We then calculate the transmission of the cavity interacting with those absorbers. For the population distributions we access, oscillators at ω_{01} remain strongly coupled to the cavity, but the splitting is slightly reduced. The other oscillators are in the weak coupling regime. Because the population is ~1% of the ground-state population, the Rabi splitting is reduced by a factor of ~10, bringing it close to the molecular and cavity linewidths. As discussed in the text, this weak coupling has measurable impact on the spectroscopy of the system. In particular, the coupling between ω_{12} and the cavity manifests as increased transmission on the low-energy edge of the spectrum after the UP decays.

Supplementary Note 4: Spectral Signature of LP Population

As discussed in the main text, LP-excited $W(CO)_6$ has no distinct spectral signature to distinguish it from the reservoir v =1 absorption because ω_{01} overlaps $\omega_{\mathrm{LP\ to\ 2}}$. Supp. Fig. 4 compares calculated ΔA and $-\log_{10}(T/T_0)$ spectra for population distributions that include both LP and reservoir v = 1 population (red) and only reservoir v = 1(blue). We again use the spectrum in Supp. Fig. 1 as the ground-state spectrum and use <1% total excitation. The spectra with and without LP population are nearly identical. We expect that LP population would lead to multiexponential decay of the slow component, but the signal-to-noise ratio of the measurements precludes us from detecting such a decay.

Supplementary Note 5: Estimating Degree of Excitation

Figure 3a in the main text shows representative calculated transient spectra at 3 and 100 ps (solid grey lines). For these calculations, we used equation S1 with time-dependent values for *A*

for the UP to v = 2 , reservoir to v = 2, and v = 0 to 1 transitions described by equations S6-S8, with superscript zeroes denoting initial amplitudes.

$$A_{UP}(t) = A_{UP}^{0} e^{\frac{-t}{\tau = 22\ ps}} \quad \text{(S6)}$$

$$A_{reservoir}(t) = A_{reservoir}^{0} e^{\frac{-t}{\tau = 170\ ps}} \quad \text{(S7)}$$

$$A_{0}(t) = A_{0}^{0} - A_{UP}(t) - A_{reservoir}(t) \quad \text{(S8)}$$

The grey curves in Fig. 3a are calculated from A_{UP}^{0} = 20, $A_{reservoir}^{0}$ = 30, and A_0^0 = 1900. These initial amplitudes correspond to an approximately 2% change compared to the initial ground state amplitude, which translates to a 1% change in population. To better match the particular data set in Fig. 3a, we used Lorentzian widths of 10 cm^{-1} for each oscillator.

Supplementary Note 6: Empty Cavity Transmission

Supplementary Figure 5 shows the transmission spectrum of an empty cavity comprising the dielectric mirrors described in the Methods section separated by a nominally 25 μm PTFE spacer (Harrick). The 140 cm^{-1} free spectral range indicates a 35 μm pathlength.

Supplementary References

1 Schwartz, T. *et al.* Polariton dynamics under strong light–molecule coupling. *ChemPhysChem* **14,** 125-131, (2013).

2 Born, M. & Wolf, E. *Principles of Optics*. (Pergamon, 1980).
